# A Complete In Vitro Toxicological Assessment of the Biological Effects of Cerium Oxide Nanoparticles: From Acute Toxicity to Multi-Dose Subchronic Cytotoxicity Study

**DOI:** 10.3390/nano11061577

**Published:** 2021-06-16

**Authors:** Adrián García-Salvador, Alberto Katsumiti, Elena Rojas, Carol Aristimuño, Mónica Betanzos, Marta Martínez-Moro, Sergio E. Moya, Felipe Goñi-de-Cerio

**Affiliations:** 1 GAIKER Technology Centre, Basque Research and Technology Alliance (BRTA), 48170 Zamudio, Spain; garciaad@gaiker.es (A.G.-S.); katsumiti@gaiker.es (A.K.); aristimuno@gaiker.es (C.A.); betanzos@gaiker.es (M.B.); 2 CIC BiomaGUNE, BRTA, 20014 Donostia-San Sebastián, Spain; erojas@cicbiomagune.es (E.R.); mmartinez@cicbiomagune.es (M.M.-M.); smoya@cicbiomagune.es (S.E.M.)

**Keywords:** Cerium oxide NPs, acute and subchronic toxicity, in vitro, pulmonary and interstitial cell lines, human airway epithelial model, air–liquid interface, aerosolized NPs

## Abstract

Engineered nanomaterials (ENMs) are of significant relevance due to their unique properties, which have been exploited for widespread applications. Cerium oxide nanoparticles (CeO_2_-NPs) are one of most exploited ENM in the industry due to their excellent catalytic and multi-enzyme mimetic properties. Thus, the toxicological effects of these ENMs should be further studied. In this study, the acute and subchronic toxicity of CeO_2_-NPs were assessed. First, an in vitro multi-dose short-term (24 h) toxicological assessment was performed in three different cell lines: A549 and Calu3 were used to represented lung tissue and 3T3 was used as an interstitial tissue model. After that, a sub-chronic toxicity assessment (90 days) of these NPs was carried out on a realistic and well-established reconstituted primary human airway epithelial model (MucilAir™), cultured at the Air–Liquid Interface (ALI), to study the long-term effects of these particles. Results showed minor toxicity of CeO_2_-NPs in acute exposures. However, in subchronic exposures, cytotoxic and inflammatory responses were observed in the human airway epithelial model after 60 days of exposure to CeO_2_-NPs. These results suggest that acute toxicity approaches may underestimate the toxicological effect of some ENMs, highlighting the need for subchronic toxicological studies in order to accurately assess the toxicity of ENM and their cumulative effects in organisms.

## 1. Introduction

In the last decades, the use of nanotechnology has revolutionized many biotechnological sectors [1]. Engineered nanomaterials (ENMs) possess unique physical, electrical, and chemical properties [2], which have been exploited for widespread applications in electronics, aerospace, medicinal drug delivery, medical devices, biosensors, engineering, bioengineering, food, and cosmetics [1,2,3,4,5,6,7,8]. The increasing use of ENMs and their consequent release into the environment [9,10,11] has raised concerns about their safety and their potential risks to human health [12,13,14,15,16].

Among these ENMs, cerium oxide nanoparticles (CeO_2_-NPs) are one of the most exploited ENMs. For instance, due to their autoregenerative cycle between two oxidation states, Ce^+3^ and Ce^+4^ [17,18], CeO_2_-NPs have been used as promising antioxidant and anti-UV agents [19]. CeO_2_-NPs have also been used as fuel catalyst additives [20], in polish surface treatment, and in cosmetics and sunscreens [21]. More recently, CeO_2_-NPs have been used as therapeutic agents to prevent blindness caused by light overexposure [22], to prevent age-related macular degeneration [23], and as anti-microbial agents by disrupting bacterial electron transport chain [24,25] and reducing the infectivity of certain viruses in vitro [26]. Despite their excellent catalytic and multi-enzyme mimetic properties [27], the potential toxicity of CeO_2_-NPs to different organisms raises concerns [28,29,30]. Thus, the toxic mechanisms of CeO_2_-NPs should be carefully and systematically investigated [27].

It has been reported that the main route of exposure to CeO_2_-NPs is through inhalation, e.g., during occupational exposure when manufacturing CeO_2_-NP-based products [31,32]. Thus, the lung is the main target organ for toxic effects after airborne CeO_2_-NPs [31,32,33,34,35] exposure.

Although animal models have been traditionally used in inhalation toxicology research, animal welfare concerns and 3R directrices encourage the use of alternative in vitro models for toxicological research [36,37,38]. In vitro models based on pulmonary cells represent excellent tools to study lung toxicity induced by exposure to ENMs. Immortalized or tumorigenic cell lines (A549, BEAS-2B, and Calu-3) are routinely used as monolayer models [39] or in co-culture with immune cells (e.g., differentiated THP-1) to study inflammatory responses induced by ENM exposure [40,41]. These in vitro models can be used both in submerged conditions or at the Air–Liquid Interface (ALI), which has been demonstrated to favor a better interaction between NPs and cells and has been considered physiologically more relevant for inhaled NPs research [32,40,42]. These models are considered useful tools for acute high-throughput screening of different air pollutants [42]. However, they are quite simplistic and do not represent an in vivo condition, since they are based on tumorigenic cells that lack inherent primary cell characteristics and do not reproduce the architecture of the lung tissue [42].

To overcome these limitations, 3D human airway epithelial models based on primary cells have already been used, as they better mimic the lung architecture and primary cells conserve original characteristics [43,44]. Currently, there are few commercially available human airway epithelial 3D models, EpiAirway^TM^ from MatTek and MucilAir^TM^ from Epithelix. MucilAir^TM^ is a reconstituted primary human airway epithelial (PHAE) model from human nasal or bronchial biopsies [45] that can be maintained in culture conditions for up to a year, allowing long-term and repeated exposures [45]. Baxter et al. [46] have demonstrated the suitability of this model for long-term exposures to toxicants. Meldrum et al. [47] used the PHAE model to assess the mid-term (up to three weeks) cytotoxicity of CeO_2_-NPs, showing that this 3D model may represent a more realistic model to predict the toxicity of inhaled particles than cell line monolayers, (which may overestimate particles’ toxicity). To the best of our knowledge, subchronic (over two months) effects of repeated exposures to CeO_2_-NPs have not yet been assessed in 3D PHAE models.

Currently, different systems have been developed to allow subchronic repeated exposure to toxicants in vitro. One of the most commonly used devices is the Vitrocell Cloud (VITROCELL Systems GmbH, Waldkirch, Germany). This device was specifically designed for exposure in ALI through the nebulization of the toxicant in a controlled atmosphere, allowing high deposition rates of the toxicant as well as robustness of results and high reproducibility [48]. This device offers the possibility of performing in vitro exposures to NPs in a much more realistic scenario.

In this context, the aim of this study was to assess acute toxicity (24 h) of CeO_2_-NPs on monocultured pulmonary (A548 and Calu-3) and non-pulmonary (3T3) cell lines and subchronic toxicity (up to 90 days) of the same nanoparticles on the physiologically relevant PHAE model exposed at the ALI through Vitrocell Cloud nebulization. This study aims to contribute to a better understanding of the cascade of acute to subchronic cellular responses in cells exposed to CeO_2_-NPs.

## 2. Materials and Methods

### 2.1. Synthesis and Characterization of CeO_2_ Nanoparticles (NPs)

CeO_2_-NPs were synthesized following the conventional gel–sol process. Briefly, commercial cerium chloride (Sigma-Aldrich, 228931, St. Louis, MO, USA) was dissolved in deionized water (0.5 M) and stirred at 400 rpm for 1 h at 60 °C in a thermostatic bath. Then, ammonium hydroxide (0.5 M) (Sigma-Aldrich, 221228, St. Louis, MO, USA) was added to the cerium chloride solution and stirred at the same conditions mentioned before for 120 min to allow NPs formation. After that, the mixture was left for 22 h at room temperature and then centrifuged, washed with deionized water, and finally heated at 110 °C to evaporate the aqueous solvent and obtain NPs as powder.

The shape and size of CeO_2_-NPs were determined by Transmission Electron Microscopy (TEM–JEM–2100F UHR, JEOL Ltd., Akishima, Tokyo, Japan). Dry powdered CeO_2_-NPs (25 µg/mL) were placed onto conducting carbon-coated copper grids for examination at the TEM. X-ray photoelectron spectroscopy (XPS–SAGE HR 100, SPECS, Berlin, Germany) was used to confirm the elemental composition and chemical state of CeO_2_-NPs.

A Zetasizer Nano ZS (Malvern Panalytical, Malvern, UK) was used to determine zeta potential and hydrodynamic size distribution through DLS analysis. The average size and polydispersity index (PDI) were determined according to ISO22412. The PDI scale was 0–1, with 0 representing a monodisperse state and 1 representing a polydisperse state. For the DLS analysis, CeO_2_-NPs (100 µg/mL) were suspended in distilled water since, due to the presence of particulate materials, no reliable measurements could be done in NP samples suspended in cell culture media.

### 2.2. Cell Culture

For the acute experiments, the murine fibroblast 3T3 cell line (CRL-1658), the lung adenocarcinoma Calu-3 cell line (HTB-55), and the alveolar epithelial adenocarcinoma A549 cell line (CCL-185) were obtained from ATCC (Wesel, Germany). The cell lines were cultured in DMEM supplemented with 10% fetal bovine serum (FBS) and 1% penicillin and streptomycin (P/S) solution (Life Technologies, Carlsbad, CA, USA), according to the manufacturer’s instructions. The culture medium was renewed every 2–3 days and the cells were subcultured when they reached 70–90% confluency.

For the subchronic experiments, fully differentiated PHAE models (MucilAir^TM^) were obtained from Epithelix Sárl (Geneva, Switzerland). The PHAE models were maintained on 24-well Transwell^®^ inserts with its own culture medium (Epithelix Sárl, Geneva, Switzerland; supplemented with 1% Amphotericin, 1% P/S, and 0.5% gentamicin). Inserts had a diameter of 6.5 mm, a growth area of 0.33 cm^2^, and a 0.4 µm pore size. Upon receipt, the PHAE models were maintained in the culture medium for at least one week prior to performing the experiments. The culture medium was renewed every 2–3 days.

### 2.3. Acute Exposures to CeO_2_-NPs

For acute exposures, 3T3, Calu-3, and A549 cell lines were seeded at 10⁴ cells/well in 96-well plates (3 × 10⁴ cell/cm^2^) and incubated for 24 h to allow confluence. Then, the culture medium was replaced by a fresh medium containing different doses of CeO_2_-NPs (10, 100 and 500 µg/mL) and cells were exposed for 24 h prior to assess CeO_2_-NPs cytotoxic effects. Before conducting the in vitro exposures, cell-free assays were carried out in order to evaluate the potential interference of CeO_2_-NPs with the toxicity assays.

### 2.4. Subchronic Exposures to CeO_2_-NPs

For subchronic exposures in ALI, 24-well Transwell^®^ inserts (Corning, 3470, Kennebunk, ME, USA) containing the PHAE model were placed into a Vitrocell^®^ Cloud exposure system (VITROCELL Systems, Waldkirch, Germany). This device is specifically designed for ALI exposure assays and consists of a 12-well chamber (8 for exposure, 1 integrated Quartz Crystal Microbalance (QCM) and 3 for control) coupled to a heating block to allow constant 37 °C temperature, and a nebulizer (Aeroneb Lab^®^, Kent Scientific, Torrington, CT, USA) on the top of the chamber. This device generates an aerosolized cloud of nanoparticles that homogeneously precipitate onto the cells due to the generated flow dynamics (vortices) after single droplet sedimentation [49]. Airway epithelia were exposed every 2 weeks during 3 months to three sublethal concentrations of CeO_2_-NPs (100 µg/cm^2^, 10 µg/cm^2^ and 1 µg/cm^2^) in order to assess their subchronic effects. After each exposure, the Transwell^®^ inserts were placed in a new 24-well plate and incubated with the culture medium.

### 2.5. Acute Cytotoxicity

Briefly, after exposures, cell viability was assessed by incubating cells with a MTT solution (0.4 mg/mL, Sigma-Aldrich, M2003-1G, St. Louis, MO, USA) for 2 h at 37 °C, 5% CO_2_. After the incubation, the insoluble formazan crystals were extracted from the cells by adding DMSO (PanReac AppliChem, A3672, Barcelona, Spain) into the wells. Absorbance was quantified at a 540 nm wavelength in a spectrophotometer reader (Varioskan™ Lux, ThermoScientific, Waltham, MA, USA). Cell viability was expressed as percentage of viability respect to untreated control cells. CdSO_4_ (Sigma-Aldrich, 383082, St. Louis, MO, USA) was used as positive control.

Apoptosis and necrosis were evaluated in exposed and unexposed cells using the Annexin-V/PI assay through flow cytometry. During the early stages of apoptosis, phosphatidylserine (PS) present on the inner leaflet of the plasma membrane is translocated to the outer layer. During apoptosis the cell membrane remains intact, whereas during necrosis the cell becomes leaky and loses its integrity. Annexin-V (Invitrogen™, A13201, Waltham, MA, USA) is a sensitive probe to detect PS on the plasma membrane of apoptotic cells. Propidium iodide (PI, Sigma-Aldrich, P4170, St. Louis, MO, USA) is a probe for discriminating necrotic cells. After NP treatment, cells were harvested and washed with an Annexin-V Binding Buffer (10 mM HEPES, pH 7.4, 150 mM NaCl, 5 mM KCl, 1 mM MgCl_2_ and 1.8 mM CaCl_2_, Sigma-Aldrich, St. Louis, MO, USA) and incubated with Annexin-V (5 µL/100 µL of cell suspension), in darkness for 30 min at room temperature. Then, the cells were washed and incubated with PI (1 µL/100 µL of cell suspension). After that, the cells were immediately analyzed in a FC-500 two laser flow cytometer (Beckman Coulter, Brea, CA, USA). Doxorubicin treated cells were used as positive control for Annexin-V or PI and non-treated cells as negative control.

ROS production was detected using the DCFH–DA dye (Sigma-Aldrich, D6883, St. Louis, MO, USA). DCFH–DA is a stable non-fluorescent, cell permeable compound that is converted to DCFH by intracellular esterases and trapped inside the cells. DCFH is then converted into the highly fluorescent 2′, 7′ dichlorofluorescein (DCF) by intracellular ROS and, upon excitation at 488 nm, emits green fluorescence proportional to the intracellular ROS levels. After treatment, the cells were harvested, washed with PBS, and incubated with DCFH–DA (5 µM) at 37 °C for 30 min in darkness. Cells were then washed, centrifuged, resuspended in PBS, and kept on ice for an immediate detection by flow cytometry using the same flow cytometer mentioned before. Doxorubicin (Sigma-Aldrich, D-1515, St. Louis, MO, USA) was used as the positive control.

### 2.6. Suchronic Effects

The effects of CeO_2_-NPs treatment on the pulmonary barrier integrity were evaluated by measuring the transepithelial electrical resistance (TEER) of the reconstituted 3D human airway epithelia according to the manufacturer’s instructions. Resistance was measured using an Epithelial Voltohmmeter (EVOM2) coupled to a STX2 chopstick electrode (World precision instruments, Sarasota, FL, USA). TEER readings were determined by subtracting the mean resistance of three inserts without cells (blank) from the recorded resistance of the airway epithelium, and subsequently multiplying the resulting value by the effective membrane surface area of the insert and expressing the results as Ω*cm^2^. As airway epithelia were cultured in ALI, 200 µL of saline solution (0.9% NaCl, 1.25 mM CaCl_2_ and 10 mM HEPES, Sigma-Aldrich, St. Louis, MO, USA) was apically added on the monolayers right before TEER measurements and then discarded at the end of the readings. TEER readings were determined every 2 days for 6 months.

The subchronic effects of CeO_2_-NP treatment on the cell viability of reconstituted 3D human airway epithelia were measured by employing the resazurin reduction method [50]. Briefly, inserts containing airway epithelia were washed with PBS and incubated with resazurin (6 µM, Sigma-Aldrich, R-7017, St. Louis, MO, USA) for 1 h at 37 °C. After incubation, samples were taken from each insert and fluorescence was read at λ_ex_ = 530 nm and λ_em_ = 590 nm in a microplate reader (Varioskan Lux, ThermoFisher Scientific, Waltham, MA, USA). Viability was expressed as percentage of viability with respect to untreated control cells. Resazurin assay was determined every 10 days for 3 months.

The effect of CeO_2_-NP treatment on the plasma membrane integrity was measured employing the LDH test CytoTox 96^®^ Non-Radioactive Cytotoxicity Assay kit, according to the manufacturer’s instructions (Promega, G1780, Madison, WI, USA). Results were expressed as percentage of damaged cells with respect to untreated control cells. LDH release was measured every 2 weeks for 3 months. Absorbance was read at λ = 490 nm in a microplate reader (Varioskan Lux, ThermoFisher Scientific, Waltham, MA, USA).

The effects of CeO_2_-NP treatment on the extra-cellular release of two inflammatory cytokines (IL-1β and TNF-α) was measured using commercially available ELISA kits (Invitrogen™ KHC3013 and KHC0012, respectively, Waltham, MA, USA), according to the manufacturer’s instructions. IL-1β y TNF-α expression were assessed in the conditioned medium after NP exposure. Results were expressed as pg/mL. Absorbance was read at λ = 450 nm in a microplate reader (Varioskan Lux, ThermoFisher Scientific, Waltham, MA, USA).

### 2.7. Statistical Analysis

In all assays, data were presented as means ± standard deviation. Normality of the data was confirmed by the Kolmogorov–Smirnoff test and the homogeneity of the variances by Levene’s test. Differences among groups were assessed by ANOVA test followed by a Bonferroni–Dunn post-hoc test. A *p*-value ≤ 0.05 was considered statistically significant. All analysis were performed using the Minitab version 16 statistic software (State Collage, PA, USA).

## 3. Results

### 3.1. Physicochemical Characterization of CeO_2_-NPs

**Figure 1:**
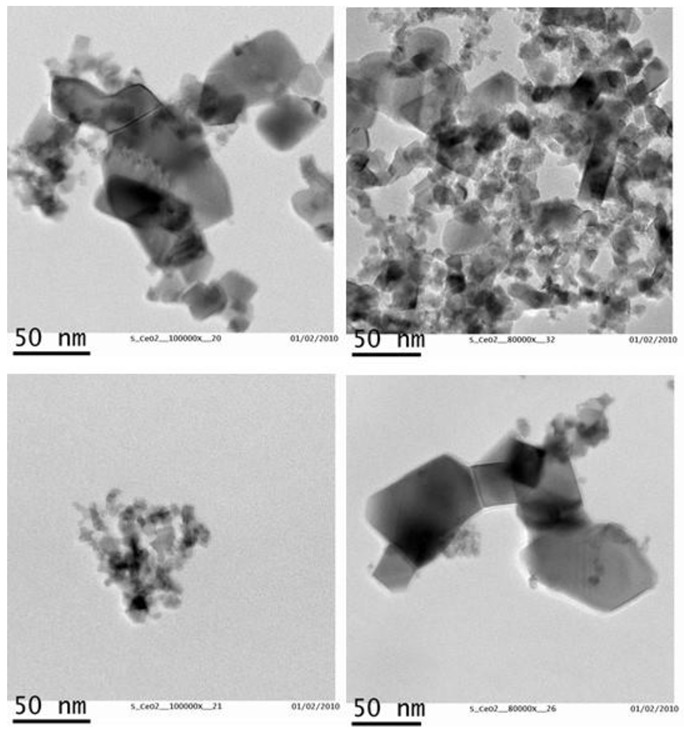
TEM images showing CeO_2_-NPs.

**Figure 2:**
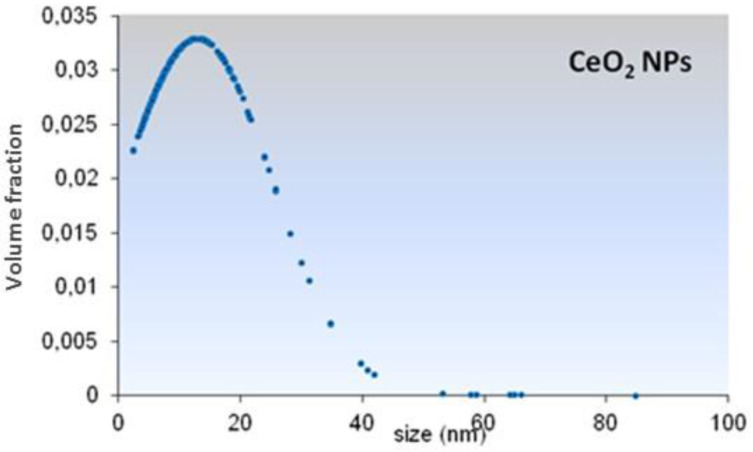
Size distribution of CeO_2_-NPs.

**Figure 3:**
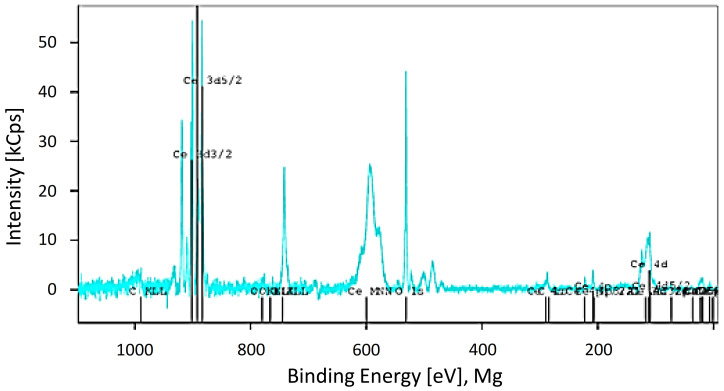
XPS spectra of Ce from CeO_2_-NPs.

CeO_2_ particles morphology and size distribution were determined by TEM. According to the TEM analysis, CeO_2_-NPs showed irregular shape with particles ranging from 4 to 64 nm (Figure 1) and a mean size of 13.04 nm ± 12.13 (Figure 2). Element analysis confirmed that particles were constituted by 21.5% Cerium and 78.5% Oxygen. No contaminants (>0.1–0.5%) were found (Figure 3).

**Figure 4:**
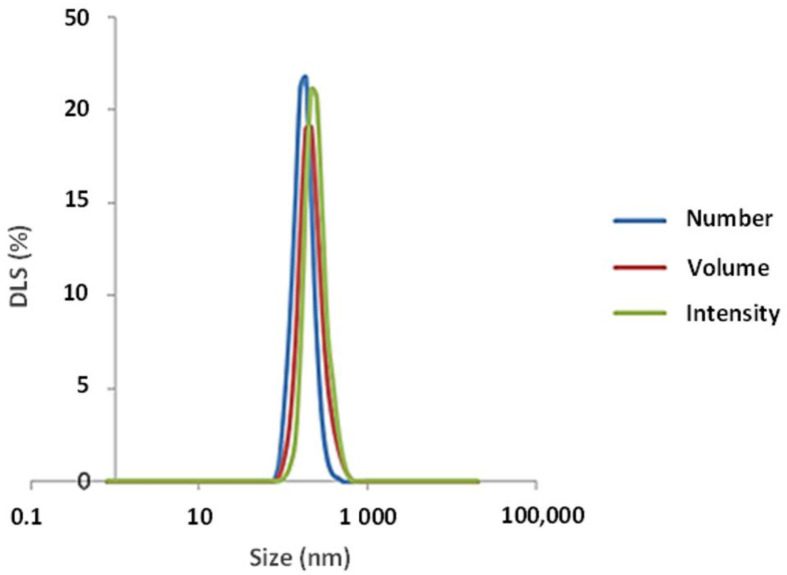
Hydrodynamic size distribution of CeO_2_-NPs suspended in water.

According to the DLS analysis, CeO_2-_NPs showed a monomodal dispersion in water (Figure 4), suggesting a homogeneous distribution. The retrieved size of particles in suspension was 44.13 nm, higher than the value obtained by TEM, indicating a possible particles aggregation. The zeta potential value of suspended particles was +36.16 mV, indicating a stable dispersion.

### 3.2. Acute Cytotoxicty

**Figure 5:**
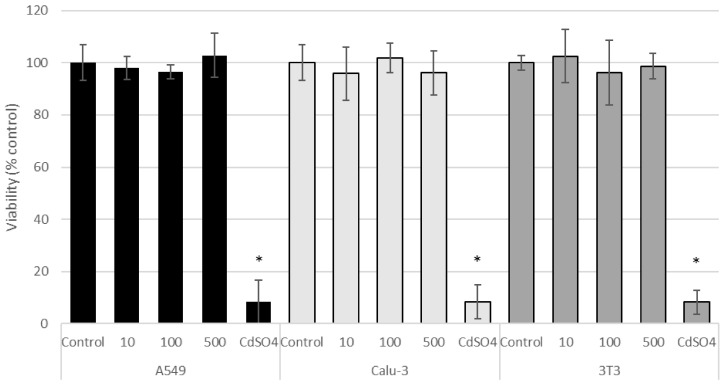
Cell viability (MTT assay) of control A549, Calu-3, and 3T3 cells, and of cells exposed for 24 h to CeO_2_-NPs (10, 100, and 500 µg/mL). Results are expressed as means ± SD of six replicates per tested condition and three independent assays (*n* = 18). Asterisks indicate significant differences with respect to the untreated control cells (*p* < 0.05).

According to the MTT assay, CeO_2_-NPs were not cytotoxic to A549, Calu-3, and 3T3 cell lines at the tested concentrations (Figure 5). CdSO_4_ (used as the positive control) significantly decreased cell viability (*p* < 0.05, Figure 5).

**Figure 6:**
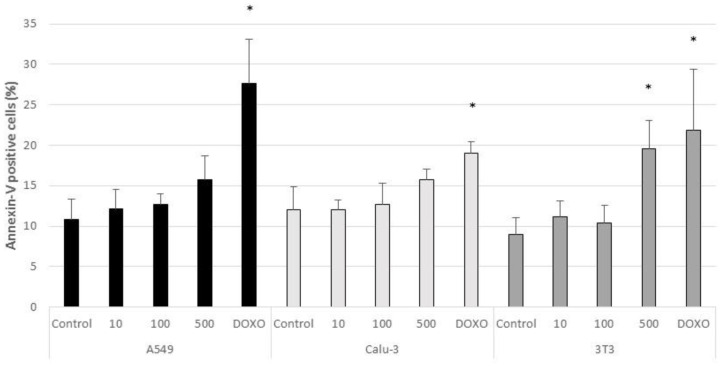
Apoptosis in control A549, Calu-3, and 3T3 cells and in cells exposed for 24 h to CeO_2_-NPs (10, 100, and 500 µg/mL), and to the positive control Doxorubicin. Results are expressed as means ± SD of six replicates per tested condition and three independent assays (*n* = 18). Asterisks indicate significant differences with respect to the untreated control cells (*p* < 0.05).

**Figure 7:**
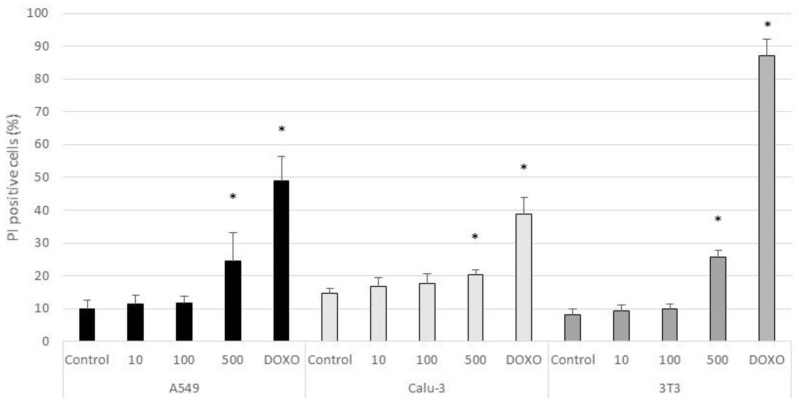
Necrosis in control A549, Calu-3, and 3T3 cells and in cells exposed for 24 h to CeO_2_-NPs (10, 100, and 500 µg/mL), and to the positive control Doxorubicin. Results are expressed as means ± SD of six replicates per tested condition and three independent assays (*n* = 18). Asterisks indicate significant differences with respect to the untreated control cells (*p* < 0.05).

The percentage of positive cells for Annexin-V and PI markers in untreated and CeO_2_-NPs treated cells are shown in Figure 6 and Figure 7, respectively. According to the results, a significant increment in the percentage of apoptotic cells (Annexin-V positive/PI negative) with respect to control cells was found in 3T3 cells treated with 0.5 mg/mL of CeO_2_-NPs (*p* < 0.05). A significant increase was also observed in the percentage of PI positive cells (necrotic cells) in all treated cell lines at 500 µg/mL (*p* < 0.05). The positive control Doxorubicin induced a significant increase of apoptosis and necrosis with respect to untreated cells in the three cell lines (*p* < 0.05).

**Figure 8:**
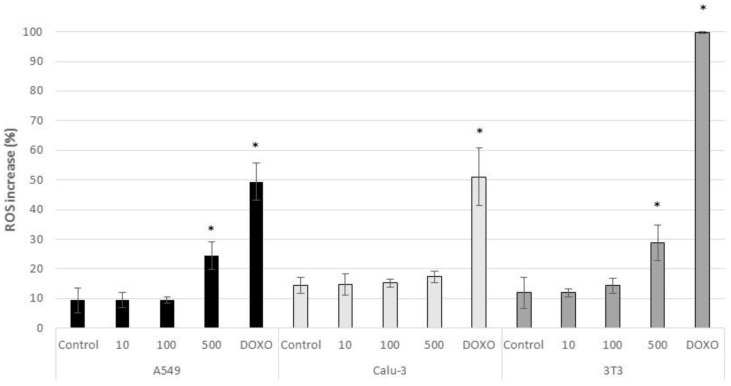
ROS production in control A549, Calu-3, and 3T3 cells and in cells exposed for 24 h to CeO_2_-NPs (10, 100, and 500 µg/mL), and to the positive control Doxorubicin. Results are expressed as means ± SD of six replicates per tested condition and three independent assays (*n* = 18). Asterisks indicate significant differences with respect to the untreated control cells (*p* < 0.05).

At tested concentrations, CeO_2_-NPs did not induce ROS production in Calu-3 cells (Figure 8). On the other hand, there was a significant increment in ROS production in A549 and 3T3 cells exposed to the highest dose assayed (500 µg/mL) with respect to untreated control cells (*p* < 0.05) (24.48 ± 4.64% in A549 cells and 28.85 ± 6.06% in 3T3 cells) (Figure 8). A significant increase in ROS production was also observed in the three cell lines treated with the positive control Doxorubicin (*p* < 0.005) (49.41 ± 6.35% in A459 cells; 51.08 ± 15.34% in Calu-3 cells and 99.71 ± 0.22% in 3T3 cells) (Figure 8).

### 3.3. Subchronic Effects

**Figure 9:**
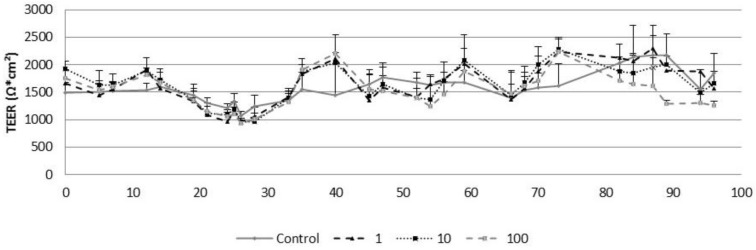
TEER values obtained in the control airway epithelia and in the airway epithelia exposed for three months to different concentrations of CeO_2_-NPs (1, 10, and 100 µg/cm^2^). Exposures were performed every two weeks for three months. Results are expressed as means ± SD of five replicates per tested condition and one assay (*n* = 5).

According to the results, TEER values ranged from 985 ± 148.79 to 2319.4 ± 292.95 Ω*cm^2^, in all treated groups and for control cells between the day 0 and the day 90 of culture. No significant differences were observed among the different groups (Figure 9).

**Figure 10:**
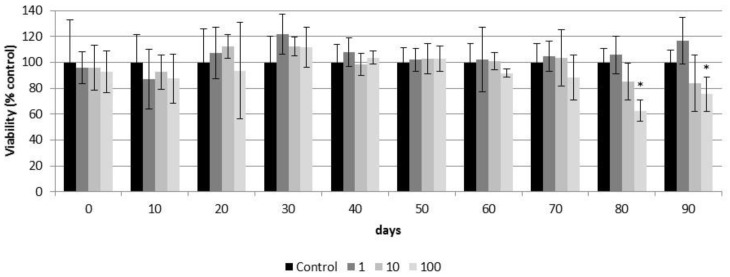
Viability (resazurin assay) of control airway epithelia and airway epithelia exposed for 90 days to different concentrations of CeO_2_-NPs (1, 10, and 100 µg/cm^2^). Exposures were performed every two weeks for three months. Results are expressed as means ± SD of five replicates per tested condition and one assay (*n* = 5). Asterisks indicate significant differences with respect to the untreated control cells (*p* < 0.05).

According to the results obtained in the resazurin assay, CeO_2_-NPs did not decrease the cell viability of airway epithelia during the first weeks (70 days) of exposure (Figure 10). However, after 80 days of treatment, CeO_2_-NPs significantly decreased the viability of airway epithelia exposed to the highest dose (100 µg/cm^2^) (day 80: 62.70 ± 8.32% and day 90: 75.5 ± 13.31%). There was no effect in cells exposed to lower doses throughout the study (Figure 10).

**Figure 11:**
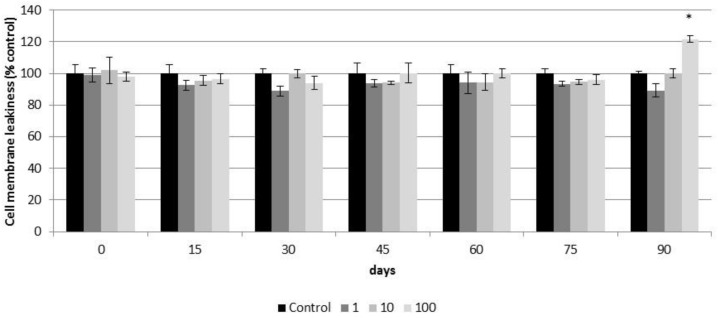
Cell membrane integrity (LDH test) of control airway epithelia and airway epithelia exposed for 90 days to different concentrations of CeO_2_-NPs (1, 10, and 100 µg/cm^2^). Exposures were performed every two weeks for three months. Results are expressed as means ± SD of five replicates per tested condition and one assay (*n* = 5). The asterisk indicates significant differences with respect to the untreated control cells (*p* < 0.05).

Based on the LDH test, CeO_2_-NPs did not affect the plasma membrane integrity of reconstituted 3D PHAE model during the first weeks (75 days) of exposure (Figure 11). At day 90 of culture, a significant increase in the LDH released was observed in airway epithelia exposed to 100 µg/cm^2^ of CeO_2_-NPs (121.65 ± 2.10%) with respect to the untreated control group (Figure 11).

**Figure 12:**
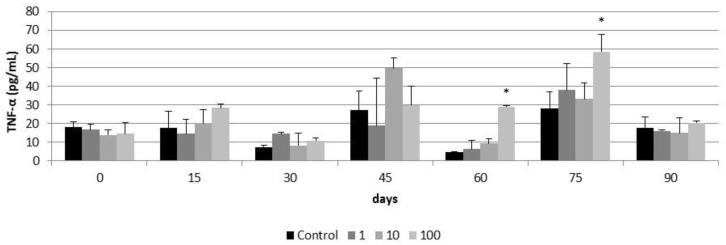
TNF-α release in control airway epithelia and in airway epithelia exposed for 90 days to different concentrations of CeO_2_-NPs (1, 10, and 100 µg/cm^2^). Exposures were performed every two weeks for three months. Results are expressed as means ± SD of five replicates per tested condition and one assay (*n* = 5). Asterisks indicate significant differences with respect to the untreated control cells (*p* < 0.05).

**Figure 13:**
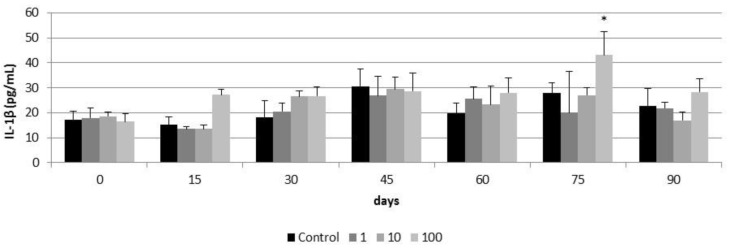
IL-1β release in control airway epithelia and in airway epithelia exposed for 90 days to different concentrations of CeO_2_-NPs (1, 10, and 100 µg/cm^2^). Exposures were performed every two weeks for three months. Results are expressed as means ± SD of five replicates per tested condition and one assay (*n* = 5). The asterisk indicates significant differences with respect to the untreated control cells (*p* < 0.05).

A significant increase in TNF-α production was observed in airway epithelia after 60 and 75 days of exposure to 100 µg/cm^2^ of CeO_2_-NPs (28 ± 0.39 pg/mL and 58.68 ± 9.36 pg/mL respectively) (Figure 12). This increase was not observed at day 90 of exposure. Lower concentrations of CeO_2_-NPs did not induce TNF-α production in airway epithelia for the 90 days of exposure (Figure 12). Similarly, a significant increase in IL-1β secretion was observed in airway epithelia exposed for 75 days to 100 µg/cm^2^ of CeO_2_-NPs (43.11 ± 9.27 pg/mL) and this increase was not observed at the day 90 of exposure (Figure 13). As for TNF-α production, lower concentrations of CeO_2_-NPs did not induce IL-1β secretion for the 90 days of exposure (Figure 13)

## 4. Discussion

As biological responses of lung cells exposed to nanoparticles depend not only on the exposure dose but also on the intrinsic properties of the NPs (e.g., size, shape, chemical composition, surface reactivity, and degree of aggregation), NPs must always be characterized before performing the toxicity assays [51,52,53] and, whenever possible the homogeneity of different batches must be ensured [54].

According to the characterization of our in-house manufactured CeO_2_-NPs, TEM analysis showed that particles ranged from 4 to 64 nm (mean size = 13.04 nm) and displayed an irregular shape. Once suspended in distilled water, DLS analysis showed particles with a hydrodynamic size of about 44 nm, with a monomodal and homogeneous distribution and high stability in the medium. The higher mean size obtained by DLS may be due to an overestimation of particle size, caused by a slight aggregation of the particles in distilled water by shear forces, as reported by other authors [55,56]. In complex media, such as a cell culture medium, aggregation might be higher due to the presence of organic molecules (e.g., amino acids). This aggregation is expected to affect the uptake and consequent toxicity of CeO_2_-NPs.

In the present study CeO_2_-NPs were not highly cytotoxic at tested concentrations but did induce cellular responses in both acute and subchronic exposures. In the acute approach, despite the absence of cytotoxicity in the MTT assay, results obtained in the more sensitive Annexin V—PI assay showed a significantly higher percentage of PI and Annexin V positive cells in the 3T3 cell line exposed to 500 µg/mL of CeO_2_-NPs, indicating that these NPs induce apoptotic and necrotic processes. Furthermore, a significant increase in ROS production was observed in both the A549 and 3T3 cell lines exposed to CeO_2_-NPs at the same concentration. Although CeO_2_-NPs are known to possess excellent antioxidant properties by scavenging free radicals, it has already been reported that they can also induce ROS production [24]. The exact mechanism by which they exhibit this oxidizing/antioxidant activity is not clearly understood, but it seems that the reason for this dual activity lies on the fact that if CeO_2_-NPs are strongly affected by the pH of the solution, then these particles can act as oxidizing or antioxidants agents [24,57]. Similar mechanisms of toxicity were reported by other authors, albeit at lower concentrations [58]. For instance, Mittal and Pandey [58] reported a concentration and time-dependent decrease of A549 viability exposed to CeO_2_-NPs at concentrations starting at 10 μg/mL. At the same concentrations, CeO_2_-NPs caused a concentration and time-dependent decrease in mitochondrial membrane potential and an increase in ROS production in the same cell model. At a lower concentration (1 μg/mL), authors reported an increase in apoptosis. The lower cytotoxicity of our CeO_2_-NPs may be related to their physical-chemical properties. Particles used by Mittal and Pandey [58] were negatively charge (−13.7 mV), whereas our particles were positively charged (+36.16). It was previously reported that negatively charged CeO_2_-NPs tend to internalize more easily than positively or neutral charged CeO_2_-NPs in cancer cell lines [54]. Thus, positively charged particles are expected to induce lower toxic effects on these cells.

Although acute toxicity studies in cell monolayers provide valuable information about the toxic effect of NPs, there is a need to use more realistic in vitro models that could better mimic lung tissue and exposure conditions and, allow for long-term subchronic and chronic evaluations [59]. Thus, the use of robust models such as PHAE models is highly encouraged. This three-dimensional model is a fully differentiated and functional human respiratory model that conserves respiratory epithelial properties such as metabolic activity, mucus production, and ciliary movement and has a life-span of up to one year [42,45,60,61]. In addition it was demonstrated through a study combining weight of evidence from proteomics, gene expression, and protein activity that this system is physiologically more suitable for repeated exposures to toxicants [46]. Therefore, after completing the assessment of the acute (24 h) cytotoxicity of CeO_2_-NPs, we proceeded to study the subchronic (3 months) cytotoxicity of repeated exposures to sublethal doses of CeO_2_-NPs in the 3D PHAE model.

In order to reproduce a more realistic exposure scenario, maintaining the conditions of temperature and humidity in physiological levels needed in the cell culture, the Vitrocell Cloud system coupled to an AeroNeb Lab nebulizer was used for exposure of the cells to CeO_2_-NPs. Other authors have already demonstrated the suitability of this system to reproduce an inhalation scenario and have demonstrated that A549 cells in vitro are able to uptake similar concentrations of CeO_2_-NPs as those taken up in vivo [62]. During the exposures, the Vitrocell Cloud system produced a very homogeneous deposition of CeO_2_-NPs onto the cells similar to previous studies [48]. Thus, a multidose experiment was performed applying three sublethal doses of CeO_2_-NPs (1, 10, and 100 µg/cm^2^) on the apical inserts every two weeks for three months. After three months of exposure to CeO_2_-NPs, the barrier integrity of the reconstituted PHAE model showed no detrimental effects, as evidenced in the TEER measurement. TEER values underwent variations throughout the three months (ranging 309–760 Ω*cm^2^), due to the constant cellular regeneration as a result of a differentiated and metabolically active epithelium composed of several cell types [44,63]. Despite the changes in cell cohesion values, TEER values always exceeded 300 Ω*cm^2^ during the exposure time. These values agree with others studies where researchers reported TEER values around 600 Ω*cm^2^ [61,64], or lower [65,66].

Based on the cell viability resazurin assay, exposure of up to 70 days to 1 to 100 µg/cm^2^ of CeO_2_-NPs showed no cytotoxic effects on the PHAE model. Similarly, up to 75 days exposure to the same concentrations of CeO_2_-NPs did not affect the plasma membrane integrity of the PHAE model. However, after 80 days of exposure to 100 µg/cm^2^ of CeO_2_-NPs, the viability of the PHAE model started to decrease. After 90 days of exposure to the same concentration of CeO_2_-NPs, the integrity of the plasma membrane of the PHAE model was compromised. The late toxic response observed in the PHAE model could be related to the protective effect of mucociliary clearance. Mucociliary clearance is a defense mechanism that protects the pulmonary system from harmful inhaled agents, including NPs. This was already reported by other authors [67] who compared the toxicity of CeO_2_-NPs on isolated Calu3 and A549 cells with the toxicity of the same particles on a PHAE model and reported that toxicity was lower in the later system, possibly due to the mucociliary defense present in the 3D model. This could indicate that the cell lines did not accurately reflect the toxic effect of the nanoparticles because they lacked the complexity of the airway tissue.

Despite the protective effect of mucociliary clearance of the PHAE model, the repeated and long-term exposure to CeO_2_-NPs possibly favored the internalization of relatively high quantities of CeO_2_-NPs, collapsing the protective system and giving rise to deleterious effects. Thus, exposures of up to 45 days to 1 to 100 µg/mL of CeO_2_ NPs did not induce inflammatory responses in the PHAE model. However between 60 and 75 days of exposure to 100 µg/mL, CeO_2_-NPs induced TNFα and IL-1β responses. TNFα and IL-1β are pro-inflammatory cytokines that activate the immune system and participate in the acute inflammatory response after exposure to a toxic agent in the pulmonary system [68]. In our study, inflammatory responses were activated after subchronic exposure to CeO_2_ NPs and prior to the decrease of plasma membrane integrity and cell viability at day 90 of exposure. As for cell viability and plasma membrane integrity, the late toxic inflammatory response in the PHAE model could be related to the protective effect of mucociliary clearance. The decrease in TNFα and IL-1β levels at day 90 of the exposure should be related to the decrease in cell viability and not due to the return to homeostasis.

Other studies have included long-term exposures (up to one month) of PHAE models to other compounds [61], demonstrating the suitability of this in vitro model as a feasible alternative to reproduce in vivo conditions and pathing the way for longer subchronic in vitro studies. Despite the fact that long-term (two years) CeO_2_-NPs exposures have been already reported in vivo [69], to the best of our knowledge, this is the first time that subchronic toxicity in vitro of CeO_2_-NPs (up to three months) have been reported. Our study confirmed the usefulness of 3D reconstituted PHAE models for long-term exposures to NPs and has helped to elucidate the subchronic effects of CeO_2_-NPs in the pulmonary epithelium. Additionally, our study has highlighted the importance of assessing the long-term effects of repeated exposure to NPs. As a recommendation for future acute and subchronic toxicity studies, a detailed dose metric analysis [70] should be performed in parallel with the in vitro assays in order to determine the expected delivered dose of NP along the exposure time, thereby corroborating biological findings and helping to support the in vitro to in vivo extrapolation of the data.

## 5. Conclusions

To conclude, acute toxicity assays based on cell lines represent useful tools for high-throughput screening of ENMs. In the case of CeO_2_-NPs, sensitive parameters (such as apoptosis–necrosis or ROS levels) are needed to elucidate the underlying mechanisms of toxicity. Nonetheless, the use of physiologically relevant cellular models, such as the reconstituted PHAE models exposed at the ALI to aerosolized NPs, represent a more realistic in vitro approach to studying the cumulative effects of long-term exposure to low doses of airborne contaminants such as CeO_2_-NPs. Thus, these in vitro systems that better mimic lung tissue and reproduce realistic exposure conditions represent valuable tools for the hazard assessment of NPs. In this particular work, we have shown that CeO_2_-NPs show a reduced toxicity in acute exposure. However, in subchronic exposures cytotoxic and inflammatory responses were observed in the human airway epithelial model after 60 days of exposure to CeO_2_-NPs. These results suggest that acute toxicity approaches may underestimate the toxicological effect of some ENMs.

## Data Availability

The source data underlying Figures are available from the authors upon request.
